# FluMob: Enabling Surveillance of Acute Respiratory Infections in Health-care Workers *via* Mobile Phones

**DOI:** 10.3389/fpubh.2017.00049

**Published:** 2017-03-17

**Authors:** May Oo Lwin, Chee Fu Yung, Peiling Yap, Karthikayen Jayasundar, Anita Sheldenkar, Kosala Subasinghe, Schubert Foo, Udeepa Gayantha Jayasinghe, Huarong Xu, Siaw Ching Chai, Ashwin Kurlye, Jie Chen, Brenda Sze Peng Ang

**Affiliations:** ^1^Wee Kim Wee School of Communication and Information, Nanyang Technological University (NTU), Singapore, Singapore; ^2^KK Women’s and Children’s Hospital (KKH), Singapore, Singapore; ^3^Tan Tock Seng Hospital (TTSH), Singapore, Singapore; ^4^Institute of Media Innovation (IMI), Singapore, Singapore

**Keywords:** mobile-health, influenza, mobile phones, application, health-care workers, surveillance

## Abstract

Singapore is a hotspot for emerging infectious diseases and faces a constant risk of pandemic outbreaks as a major travel and health hub for Southeast Asia. With an increasing penetration of smart phone usage in this region, Singapore’s pandemic preparedness framework can be strengthened by applying a mobile-based approach to health surveillance and control, and improving upon existing ideas by addressing gaps, such as a lack of health communication. FluMob is a digitally integrated syndromic surveillance system designed to assist health authorities in obtaining real-time epidemiological and surveillance data from health-care workers (HCWs) within Singapore, by allowing them to report influenza incidence using smartphones. The system, integrating a fully responsive web-based interface and a mobile interface, is made available to HCW using various types of mobile devices and web browsers. Real-time data generated from FluMob will be complementary to current health-care- and laboratory-based systems. This paper describes the development of FluMob, as well as challenges faced in the creation of the system.

## Introduction

Seasonal influenza affects nearly 20–25% of the Singapore population (1). The all-cause mortality attributable to influenza stands at 14.8 per 100,000 person-years, making the burden comparable to other temperate countries (2). Globally, it is estimated that there were approximately 284,500 respiratory and cardiovascular deaths associated with the 2009 influenza pandemic (3). Due to Singapore’s geographical location, pandemic threats from respiratory infectious diseases continue to persist, e.g., avian influenza A subtype viruses (H5N1 and H7N9) in Shanghai, China, and the Middle East respiratory syndrome coronavirus in the Middle East, in addition to seasonal influenza. The true impact of influenza often stretches beyond the viral illness itself and contributes to other disease burden by causing complications in patients with preexisting conditions (i.e., cardiovascular diseases or cardiopulmonary disease).

Economic modeling has recently demonstrated that the treatment-only strategy for influenza resulted in a mean number of 690 simulated deaths, 13,950 hospital days, an equivalent of 2.5 million workdays lost, and a mean economic cost of USD$469.8 million per year (4). Southeast Asia is acknowledged as a hotspot for emerging infectious diseases (5), and Singapore—as a travel and health hub of the region—faces a constant risk of pandemic outbreaks. The 2003 severe acute respiratory syndrome outbreak proved to be a huge burden on Singapore’s economy, costing US$570 million and resulting in unprecedented rates of unemployment at 5.5% (6, 7). Existing and potential threats highlight the importance of having robust surveillance and health communication systems present, which can forewarn people, detect unusual signals and provide health education in an efficient and cost-effective manner.

Given the absence of an efficient surveillance system that addresses challenges within hospitals in Singapore, this paper reports the design and development of a prototype integrated mobile-health participatory influenza surveillance system entitled FluMob. Following a review of literature on information and communication technology (ICT) approaches to addressing influenza tracking and surveillance, we describe FluMob’s architecture, followed briefly by the methodologies used to recruit and retain users. Finally, we present the challenges the research team faced in the various phases of the implementation of the intervention, and lessons learnt, which will be useful to public health researchers and practitioners involved in similar initiatives or interventions in the future.

## Related Literature

Participatory epidemiology (PE) is a concept that has increasingly been used in health surveillance in recent years. It uses community involvement to improve the understanding and control of diseases and was most prominently brought to attention by work conducted in Africa investigating animal health from information gathered by local farmers (8).

With the proliferation of Internet and mobile phone usage, ICT has played a significant role in the development of PE for disease surveillance, health monitoring, and information sharing; enabling both individuals at the point of care and stakeholders such as health authorities and health providers to be directly linked to the communities they served. Platforms such as “*Outbreaks Near Me*” and “*Ushahidi*” have been effective in optimizing the collaboration between ICT and health surveillance (9). Communication through ICT such as mobile phone messaging has also been used to influence health behaviors by encouraging healthy eating and exercise (10), adhering to medication recommendations (11), and promoting the cessation of smoking (12). With the increase of mobile phone usage, health-care workers (HCWs) in developing countries are now able to effectively collect health data in a quick and economical way (13).

Collecting real-time surveillance data provide the foundation for any pandemic preparedness program, but current approaches continue to rely on traditional methods with minimal use of new technology or social engagement. For example, existing infrastructure for influenza surveillance and epidemiology are focused on health-care institutions providing clinical reports of acute respiratory infections as well as laboratory-based confirmed influenza cases (14). These methods usually rely on the symptomatic person visiting a health-care facility, and such systems can be made less efficient by poor health-seeking behavior and delays in disease notifications. Despite their strengths, the setup and maintenance of these systems can be costly, particularly in developing countries (13). During the 2009 H1N1 pandemic, public health bodies worldwide faced difficulties and delays in ramping up such traditional surveillance systems (15).

To address the limitations of routine surveillance systems during pandemic H1N1 in 2009, a number of countries such as the UK urgently developed Internet-based systems to be used by the public (16). These have shown good results and continue to be used for routine seasonal influenza. Other approaches have included the development and use of population web searches on influenza-related terms to help predict an outbreak of infectious disease (17). However, despite early acclaim during pandemic outbreaks, systems such as *Google Flu trends* have been shown to be too sensitive to media reports, resulting in difficult to control biases, particularly during normal influenza seasons (18, 19).

More recently, Lwin et al. (20) reported the application of the PE approach to the conceptual and technological development of a mobile-based crowd-surveillance application called Mo-Buzz for use by public health inspectors and the general public to address dengue outbreaks in Sri Lanka. Other similar initiatives have adopted this approach to bolster the public health management of asthma, and natural disasters such as earthquakes (9). While most of these efforts send health alerts or enable people to report disease experiences, they offer little by way of telling the user how exactly to prevent or protect oneself from the outbreak. Singapore’s pandemic preparedness framework—confronted by a significant influenza burden and looming threat of emerging infectious diseases—can be strengthened by utilizing the mobile-based PE approach and improve upon existing ideas by addressing clear gaps (such as a lack of health communication).

The rapid development and innovation of new and affordable tablet devices, digital applications, and geographic information systems have become easily accessible to the Singaporean population, with nearly 90% smartphone penetration. Therefore, Singapore is best positioned to spearhead the development of this public health innovation in the region and to scientifically evaluate its impact on population groups at risk from influenza. These technologies can be integrated to design an innovative dynamic system where health authorities obtain real-time epidemiological and surveillance data from HCWs within Singapore who report disease incidence using smartphones.

The data generated from such a system with its significant time advantage could detect clusters of diseases and could be used as early warning signals for emerging influenza outbreaks within the hospital context, allowing public health authorities to initiate further investigations. The above literature emphasizes how real-time surveillance has become increasingly important in investigating infectious diseases such as influenza, which remains a social and economic burden. Given that smartphones are becoming more widespread in developing countries due to decreasing costs and increasing availability, pandemic preventative programs need to focus on integrating social media to streamline influenza surveillance, treatment, and health communication.

## Development of FluMob

### Technical Specifications

**Figure 1:**
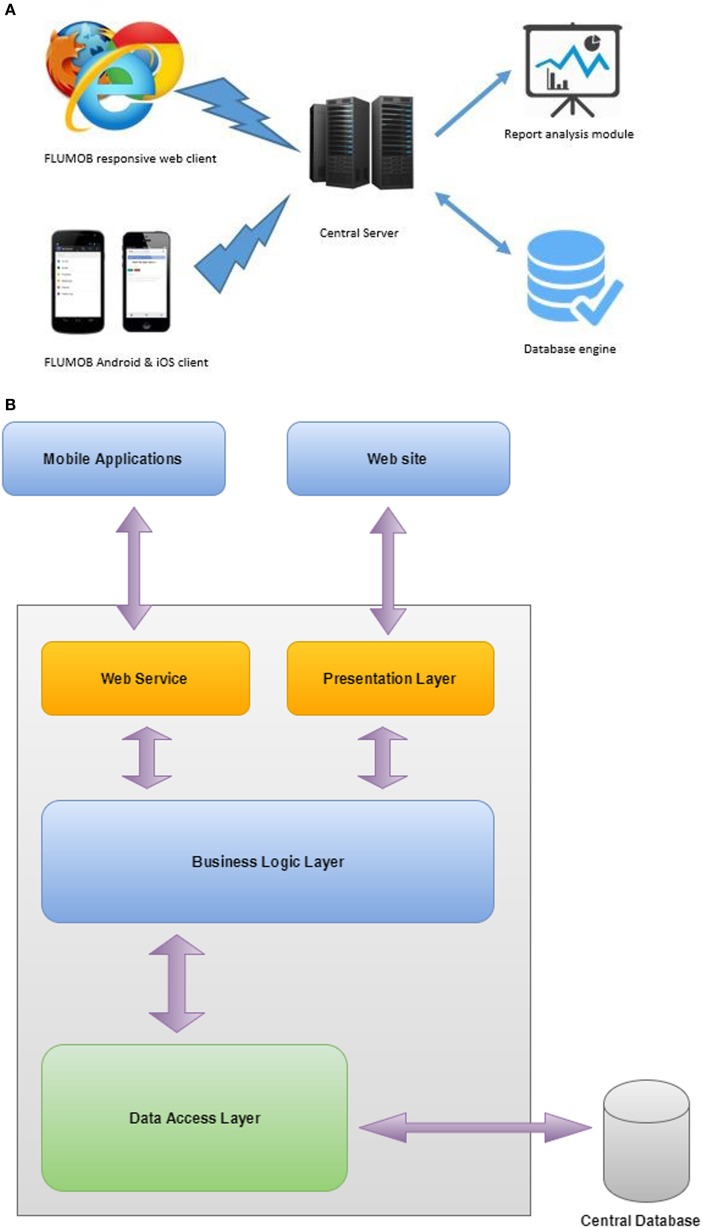
**FluMob architecture: (A) conceptual model and (B) system architecture**.

The FluMob system blends ubiquitous access to the Internet, and the simple portability of mobile phones to create a digitally integrated syndromic surveillance system. The system, integrating a fully responsive web-based interface and a mobile interface, is made available to HCWs using various types of mobile devices and web browsers. The ease and convenience in using application software on their mobile phones will allow users to provide reports of non-specific syndromes such as influenza-like illness (ILI) on a weekly basis. The near real-time data generated from the system will be complementary to current health-care- and laboratory-based systems in assisting with streamlining hospital outbreak response among HCWs and informing vaccine policy. Figure 1 shows the overall system architecture of FluMob. The application supports two mediums of data input (web browsers and mobile phones) that are fed into a central server and are subsequently generated as reports to be analyzed.

The FluMob application consists of mobile operating systems (Android and iOS) and a responsive web portal. These applications are integrated into a central database using common web services. Central servers hold the business logics related to the FluMob application and the report analysis module. Once users are registered in the system, they have to log in with user identifications and passwords. There are no identified constraints in the application, and it is a simple, user-friendly process. All required data will be stored in an encrypted manner for security and confidentiality purposes.

### Operating Environment

The operating environment of FluMob can be divided into two components: *software environment (SE)* and *hardware environment (HE)*.

The SE is the collection of software required to operate the application, and those used in the FluMob application are Windows server 2008 R2, Apache/2.4.17, PHP Version 5.5.30, MySQL 5.6, Android studio, and xCode for iOS development.

The HE refers to the set of hardware required to deploy the application. The FluMob central server is configured with Core2 Intel Xeon Processor with four cores, 8 GB of random-access memory, and 500 GB of storage space. The main server supports any number of web clients. Based on the initial system prototype, more than 100 clients are expected, and the system was tested with 500 dummy clients. The system supported 100 concurrent users without any technical malfunctions. The maximum number of sever connections was restricted to 100 connections, which proved to be sufficient, as database servers will be configured to allow connection pooling. There are no specific security mechanisms added to the client application, but predefined private keys to communicate with central servers have been implemented.

## Participant Engagement

**Figure 2:**
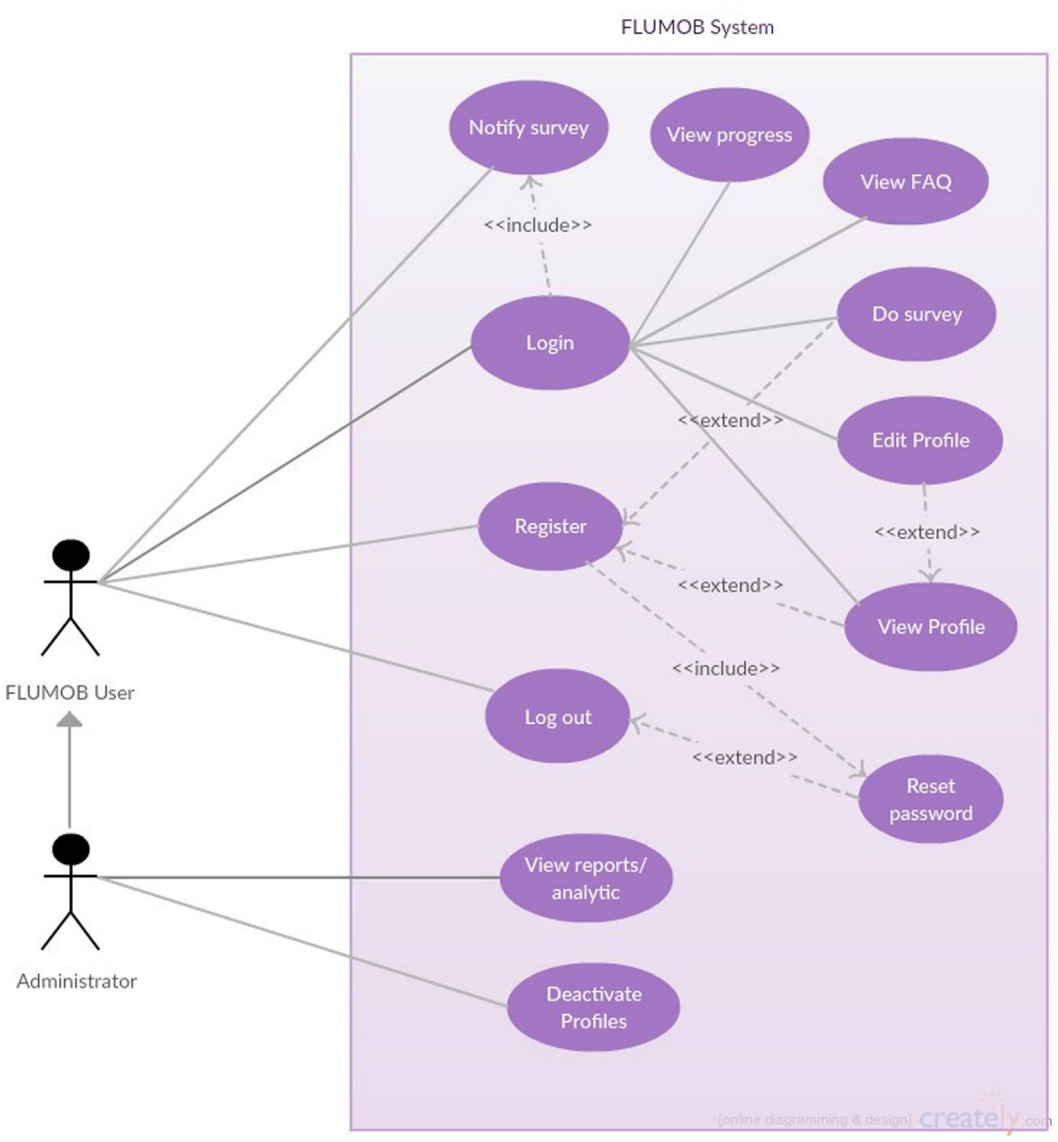
**Use case diagram for FluMob**.

Figure 2 shows the use case diagram for FluMob. New users are first required to register with the system to define their profiles. The login system provides functionality for users to view the FAQs associated with the system and allows them to make changes to their profile information and reset their passwords. At a predetermined schedule, users are notified to log into the system and carry out the routine survey. At any time, users can view all their past survey returns and changes over time. The accumulated survey results are analyzed and made available to the administrator of the system for further actions.

**Figure 3:**
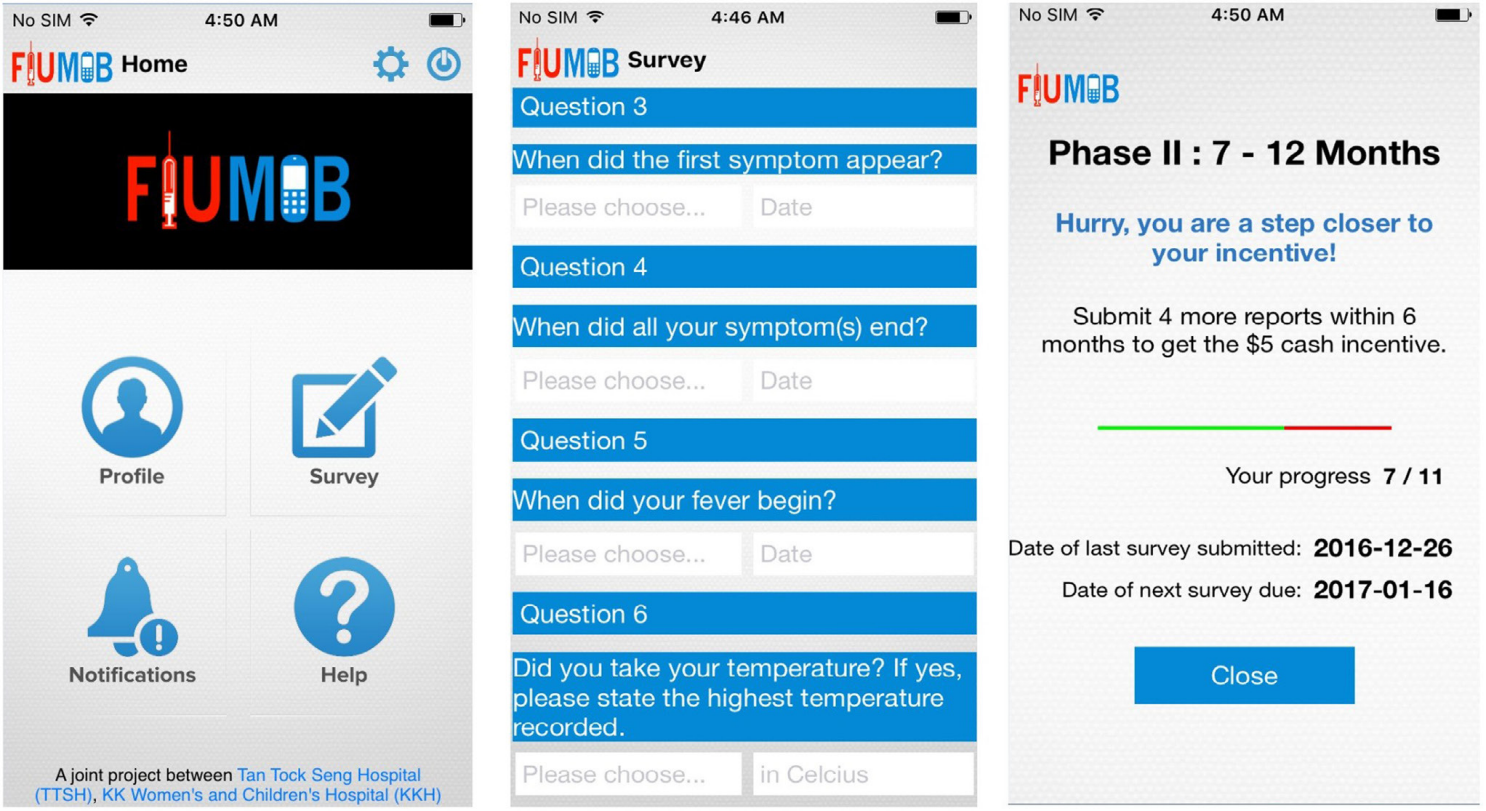
**Screenshots of the FluMob mobile application**.

The FluMob system is being tested and used by consenting participants from Tan Tock Seng Hospital (TTSH) and KK Women’s and Children’s Hospital (KKH). TTSH has a Communicable Disease Centre and is the designated hospital to handle and manage outbreaks of novel diseases. KKH is a women’s and children’s hospital, with a large inpatient and outpatient pediatric patient workload. The research is being conducted using standard research practice and ethics guidelines. An optimal sample size of 278 was calculated for the study’s statistical validation representing the health-care workforce using G*Power analysis (21). However, factoring in attrition rates, the researchers aim to recruit 700 HCWs. Participants, who include clinical and non-clinical HCW across these two hospitals, are required to be no less than 21 years old, and own smartphones installed with either iOS or Android software. Hospital staff at all departments were invited to download the app *via* mass emails. Upon responding, users are given a link to the relevant software app store to download the free app. Once the app is loaded on the mobile phone, each user is first asked to register by filling a form capturing demographic, lifestyle details, and medical history. Figure 3 shows the screenshots of the mobile application on a typical screen.

Clinical and social scientists from collaborating institutions developed and collated a range of questions to capture data relating to HCW demographics, lifestyle, influenza virus symptoms, and prevention. FluMob registration requires participants to fill in a form capturing demographic details (e.g., date of birth, sex, and ethnicity), workplace information (e.g., hospital name, job category, and department), information about family (e.g., how many people in different age groups), lifestyle behaviors (e.g., mode of transport to work and frequency of eating at food centers), medical history (e.g., vaccination records and disease profiles), as well as technology use and acceptance (e.g., usage of mobile phone, Internet, and mobile applications). The questions serve as a baseline for researchers to understand the lifestyle patterns and technology consumption among local HCWs. Descriptive analyses could potentially assist in the development of policies for disease monitoring and preventive measures. The data collected at registration can also be used for analytics at a later stage to identify any potential relationship between demographics, lifestyle behaviors, medical history, and vulnerability to influenza.

Health-care workers are prompted to submit weekly health reports on whether they have ILI symptoms, a dichotomous “yes-or-no” question is first presented to the users to capture the presence of ILI symptoms after they have chosen their ward/location of duty. If users answer “no,” they will then receive a “thank you” note for submission and can immediately resume their daily work tasks or activities. Conversely, when users have declared having ILI symptoms, they will be asked to specify their symptoms from a list, which includes fever, cough, muscle/joint pain, vomiting, diarrhea, and others. After which, users will then need to provide further information regarding the illness, such as the date of onset and end of symptoms, body temperature, whether they have fever, medical services visited, medication taken as well as some medical leave-related questions. Finally, they will be asked to rate their health status on the day itself on a scale of 0–100.

This component was designed to enhance surveillance efforts with real-time information about ILI episodes among the clinical and non-clinical staff in both hospitals. The reports are submitted on mobile phones or web browsers to assist the research team in detecting potential influenza outbreaks within the hospital. Users are provided with incentives after submitting a certain amount of reports. As soon as a user has submitted the report, the information is stored in a data repository, which allows clinicians and researchers to gather real-time crowd-sourced information for clinical analytics so as to inform strategies for disease surveillance, prevention, and management.

## Progress and Status

The Android version of the application was introduced to the health workers at TTSH and KKH in May 2016, and saw over 50 HCWs from TTSH signing up for the study within the first week. The iOS version was launched later in June 2016, and there are currently more than 200 iOS users who have installed the FluMob application. At this stage, the team has steadily recruited almost 700 participants. Of these, approximately 50% are regularly submitting weekly reports.

## Challenges and Learning Experiences

A number of challenges were faced in the development and implementation of the system. This section will look at the challenges faced, and how they were addressed and resolved by the team. The first trial was encountered during the development phase of the application. The most recent data available (22) show that the Android (i.e., Samsung S-series) software for mobile phones dominates the Singaporean market, holding 65.58% of the market share, whereas iOS (i.e., Apple iPhones) holds 27.24%.

Therefore, the technical expertise of the research team focused only on the development of Android-based applications and outsourced the development of the iOS version to an external development specialist. Due to the demands of the project and other unforeseen circumstances, the study was first launched only with the Android application, and interested IOS individuals had to be put on waiting list for more than a month. When the IOS version was finally released and individuals on the wait list were re-contacted, a lot of the initial interests had waned leading to only 75% of them being successfully recruited into the study.

To prevent the coding and programming issues described earlier, a platform where both Android and iOS mobile phone applications can be developed simultaneously can be considered in the future. A software called Appcelerator Titanium (23) can be used to create a full-featured iOS application using JavaScript and can automatically convert the JavaScript code into Objective-C code, which is a requirement of coding for iOS mobile applications. Creating the Android version of the same application is also simplified as the Titanium software will convert the JavaScript code into Java and create an application suitable for the Android Marketplace.

The second challenge pertained to the type and number of survey questions that were to be included in both the registration and the weekly reports sections of the FluMob application. The researchers were faced with the arduous task of filtering through numerous survey questions that effectively measured demographic variables (i.e., socioeconomic status, sex, and age) and overall health of the participant (i.e., smoking status). Sifting through previously published peer-reviewed literature took time, and numerous meetings were required to settle on the questions which were to be included.

This issue was resolved by meeting frequently, and by using scales that have been previously tested and established in their efficacy at measuring ILI symptoms. The team also resolved differences in opinion in an objective, evidence-based manner, which allowed for more empirical formulation of survey questions. The question list was pilot tested on a small sample (*N* = 10) of participants from TTSH. This allowed for feedback to be collected and amendments made prior to the large-scale implementation of the application.

**Figure 4:**
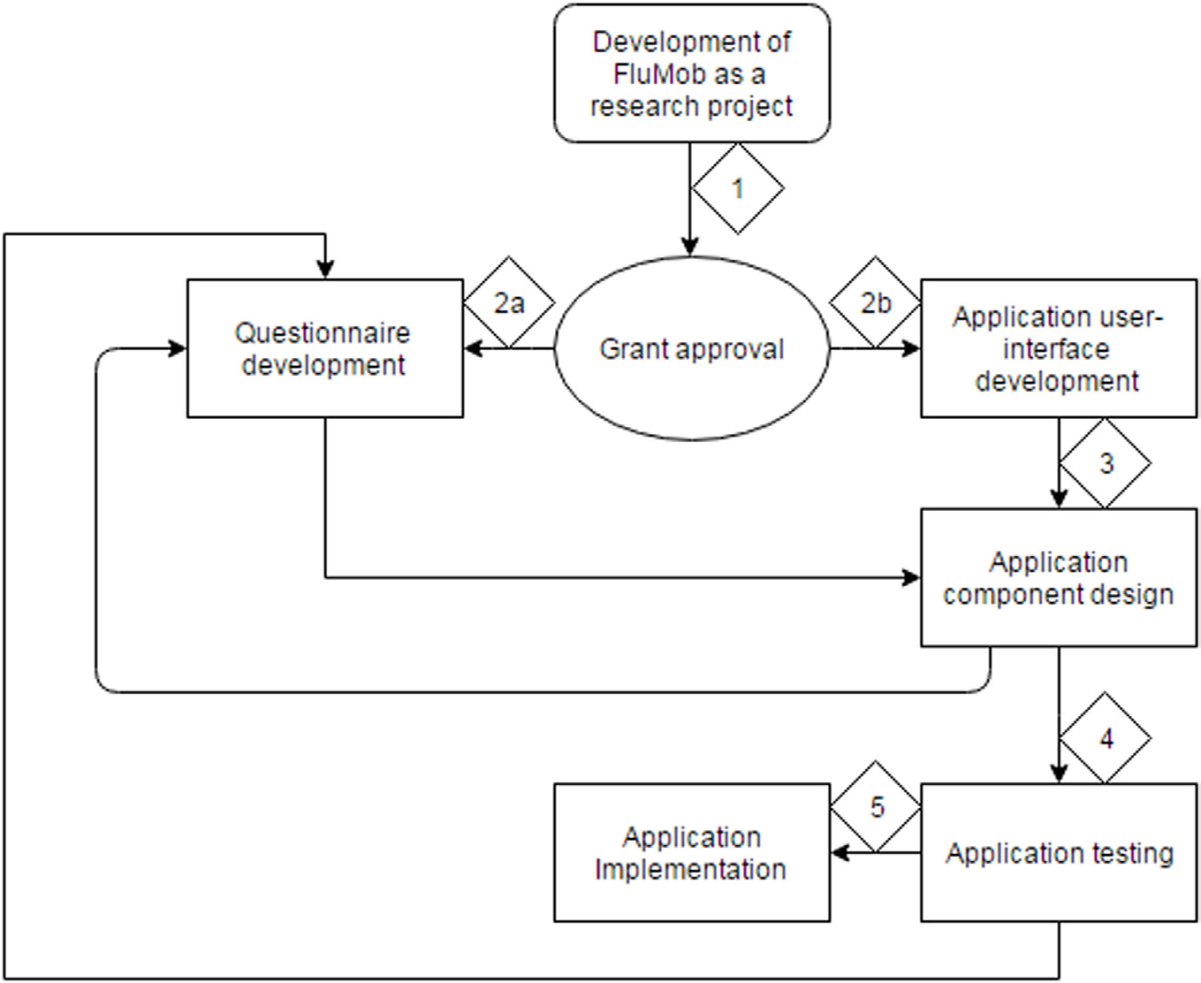
**Flowchart describing the process of the FluMob application development**.

The final challenge arose in the form of inter-organizational and transdisciplinary research. The research team comprises of clinician scientists, social scientists, and research engineers, hailing from several different institutions; Nanyang Technological University, KKH, TTSH, National University of Singapore, and National Public Health Laboratory. Figure 4 shows the flowchart visualizing the work flow involved in developing the FluMob application.

In Figure 4, the diamond-shaped boxes with numerical values describe the order of the process. As shown in the chart, the idea for the development of the application is the first step, after which grant writing and submission ensue. After approval, the team splits into two groups; the clinical/social science groups (2a) and the research engineering group (2b). After the development of the user interface of the application, the research engineer team should bring the application into its testing phase (3). However, frequent revisions to the application pertaining to both the design and the survey questions were made by the clinical/social science team. This resulted in multiple phases of component design and testing (4), which inherently delayed the implementation of the application (5).

The research team resolved the issue of constant iterations of the survey by completing full scale testing within 1 week and freezing any changes that could be made to the application a week prior to launch. The final version of the survey was fully agreed upon by both clinical and social scientists and allowed for a measurement of the full spectrum of variables that permitted all the research hypotheses to be tested effectively. The nature of having experts of varied specializations gave project a larger research scope, limited to not just social science or clinical science. This is an example of how transdisciplinary research can be both an advantage and a disadvantage to the implementation of such a research project.

## Discussion and Future Development

The completion of the study period will see detailed data analysis, which includes an analysis of the weekly reports and cases identified for follow-up. The registration questions will serve as a baseline for researchers to understand the lifestyle patterns and technology consumption among local HCWs. Descriptive analyses will also yield valuable data and could potentially assist in the development of policies for disease monitoring and preventive measures. The data collected at registration can also be used for analytics at a later stage to identify any potential relationship between demographics, lifestyle behaviors, medical history, and vulnerability to influenza.

At the next stage, our plan is to incorporate health education messaging and communication. The present system allows for users to select the option to enable or disable notifications and avoids broadcasting of messages, instead electing to personalize reminder messages for each user. The research team wants to build on this and is considering including, in a subsequent version of FluMob, a health education messaging service that will send out health educational messages to users when they report having flu-like symptoms. For example, if a user were to report fever as a symptom, a notification would be sent to the user to encourage them to wear a mask, avoid contact with others, or to see a doctor. Two areas of academic inquiry are being considered by the research team; the first tests the efficacy of more tailored messages, and the second studies the effects of various modalities of communicating health messages.

The FluMob study is currently under deployment with participants in both hospitals where data are being collated, the results of which will be analyzed in the near term future. At of the time of writing, recruitment numbers are still increasing, and weekly influenza reports from HCWs are being steadily submitted. The research team is presently building upon the knowledge gained to create a novel integrated syndromic surveillance system for general public use, which they hope will further address the gaps in disease prevention on a wider national and regional scale, and streamline influenza surveillance to reduce the burden of emerging infectious diseases.

## Ethics Statement

This study was carried out in accordance with the recommendations of DSRB, National University of Singapore with written informed consent from all subjects. All subjects gave written informed consent in accordance with the Declaration of Helsinki. The protocol was approved by the National University of Singapore.

## Author Contributions

ML, BA, CFY, PY, and SF were involved in the conceptualization of the paper and the overall editing. KJ, AS, and KS wrote the main sections of the paper. HX and CJ were involved in data collection in their respective hospitals. UJ, AK, and KS were involved in the technical development of the application. SC was the overall coordinator for the project.

## Conflict of Interest Statement

The authors declare that the research was conducted in the absence of any commercial or financial relationships that could be construed as a potential conflict of interest.
